# Certain Subclasses of Analytic Functions with Complex Order

**DOI:** 10.1155/2013/958796

**Published:** 2013-10-29

**Authors:** A. Selvam, P. Sooriya Kala, N. Marikkannan

**Affiliations:** ^1^Department of Mathematics, VHNSN College, Virudhunagar 626001, India; ^2^Department of Mathematics, Government Arts College, Melur 625106, India

## Abstract

Two new subclasses of analytic functions of complex order are introduced. Apart from establishing coefficient bounds for these classes, we establish inclusion relationships involving (*n*-*δ*) neighborhoods of analytic functions with negative coefficients belonging to these subclasses.

## 1. Introduction

Let *𝒜*(*n*) denote the class of functions of the form

(1) $$\begin{array}{c} f\left(z\right)=z-\overset{\infty }{\underset{k=n+1}{\sum }}a_{k}z^{k}\;\left(a_{k}\geq 0,n\in \mathbb{N}:=\left\{1,2,3,\ldots \right\}\right), \end{array}$$

which are analytic and univalent in the open disc

(2) $$\begin{array}{c} \Delta =\left\{z\in \mathbb{C}:\left|z\right|<1\right\}. \end{array}$$

A function *f*(*z*) ∈ *𝒜*(*n*) is star-like of complex order *b*, denoted as *f*(*z*) ∈ S*(*b*) if and only if it satisfies

(3) $$\begin{array}{c} ℜ\left\{1+\frac{1}{b}\left(\frac{zf^{\prime }}{f}-1\right)\right\}>0\;\left(z\in \Delta \right). \end{array}$$

A function *f*(*z*) ∈ *𝒜*(*n*) is convex of complex order *b*, denoted as *f*(*z*) ∈ *C*(*b*) if and only if it satisfies

(4) $$\begin{array}{c} ℜ\left\{1+\frac{1}{b}\left(\frac{zf^{\prime \prime }}{f^{\prime }}\right)\right\}>0\;\left(z\in \Delta \right). \end{array}$$

These classes, S*(*b*), and *C*(*b*) are introduced and studied by Nasr and Aouf [1] and Wiatrowski [2].

For the two functions *f*_*j*_(*j* = 1,2) given by

(5) $$\begin{array}{c} f_{j}\left(z\right)=z+\overset{\infty }{\underset{k=2}{\sum }}a_{k,j}z^{k}\;\left(j=1,2\right), \end{array}$$

the Hadamard product or convolution, denoted by (*f*_1_∗*f*_2_)(*z*), is given by

(6) $$\begin{array}{c} \left(f_{1}*f_{2}\right)\left(z\right)=z+\overset{\infty }{\underset{k=2}{\sum }}a_{k,1}a_{k,2}z^{k}. \end{array}$$

Given *f*(*z*) of the form (1) and *δ* ≥ 0, we define *n*-*δ* neighborhood of a function *f* ∈ *𝒜*(*n*) as

(7) $$\begin{array}{c} N_{n,\delta }\left(f\right)=\left\{g\in 𝒜\left(n\right)\;|\;\overset{\infty }{\underset{k=n+1}{\sum }}k\left|a_{k}-b_{k}\right|\leq \delta \right\}. \end{array}$$

In particular, for the identity function *e*(*z*) = *z*,

(8) $$\begin{array}{c} N_{n,\delta }\left(e\right)=\left\{g\in 𝒜\left(n\right)\;|\;\overset{\infty }{\underset{k=n+1}{\sum }}k\left|b_{k}\right|\leq \delta \right\}. \end{array}$$

The concept of Neighborhood *N*_*n*,*δ*_ of a function *f* is introduced and studied by Ruscheweyh [3] and extended further by Silverman [4].

For complex numbers *α*_1_, *α*_2_,…, *α*_*q*_ and *β*_1_, *β*_2_,…, *β*_*s*_ (*β*_*j*_ ∈ *ℂ*∖*𝒵*_0_^−^; *𝒵*_0_^−^ = {0, −1, −2,…} for *j* = 1,2,…, *s*), we define the generalized hypergeometric function _*q*_ *F*_*s*_(*α*_1_, *α*_2_,…, *α*_*q*_; *β*_1_, *β*_2_,…, *β*_*s*_; *z*) as

(9) Fsq(α1,α2,…,αq;β1,β2,…,βs;z)=∑k=0∞(α1)k(α2)k⋯(αq)kzk(β1)k(β2)k⋯(βs)kk!, (q≤s+1;q,s∈ℕ0:=ℕ∪{0};z∈U),

where *ℕ* denotes the set of all positive integers and (*x*)_*k*_ is the Pochhammer symbol defined in terms of gamma functions as

(10) (x)k=Γ(x+k)Γ(x)={1if  k=0x(x+1)⋯(x+k−1)if  k∈ℕ.

Corresponding to the function *g*_*q*,*s*_(*α*_1_, *β*_1_; *z*) defined by

(11) gq,s(α1,β1;z)=z qFs(α1,α2,…,αq;β1,β2,…,βs;z)

recently in [5], an operator *𝒟*_*λ*,*μ*_^*m*^(*α*_1_, *β*_1_)*f*(*z*) : *𝒜* → *𝒜* is defined by

(12) 𝒟λ,μ0(α1,β1)f(z)∶=f(z)∗gq,s(α1,β1;z),𝒟λ,μ1(α1,β1)f(z)∶=(1−λ+μ)(f(z)∗gq,s(α1,β1;z))+(λ−μ)z(f(z)∗gq,s(α1,β1;z))′+λμz2(f(z)∗gq,s(α1,β1;z))′′,𝒟λ,μm(α1,β1)f(z)∶=𝒟λ,μ1(𝒟λ,μm−1(α1,β1)f(z)),

where 0 ≤ *μ* ≤ *λ* ≤ 1 and *m* ∈ *ℕ*_0_. By the above definition, it is easy to note that

(13) 𝒟λ,μm(α1,β1)f(z)z+∑k=2∞[1+(k−1)(λ−μ+kμλ)]m×(α1)k−1(α2)k−1…(αq)k−1(β1)k−1(β2)k−1…(βs)k−1(k−1)!akzk.

Let us take for convenience that

(14) $$\begin{array}{c} B_{k}=\frac{{\left(\alpha_{1}\right)}_{k-1}{\left(\alpha_{2}\right)}_{k-1}\ldots {\left(\alpha_{q}\right)}_{k-1}}{{\left(\beta_{1}\right)}_{k-1}{\left(\beta_{2}\right)}_{k-1}\ldots {\left(\beta_{s}\right)}_{k-1}\left(k-1\right)!},\\ C_{k}=1+\left(k-1\right)\left(\lambda -\mu +k\mu \lambda \right). \end{array}$$

Hence, we have

(15) $$\begin{array}{c} {𝒟}_{\lambda ,\mu }^{m}\left(\alpha_{1},\beta_{1}\right)f\left(z\right)=z+\overset{\infty }{\underset{k=2}{\sum }}C_{k}^{m}B_{k}a_{k}z^{k}. \end{array}$$

For suitable values of *α*_*i*′*s*_, *β*_*j*′*s*_, *q*, *s*, *m*, *λ*, and *μ*, we can deduce several operators such as Sălăgean derivative operator [6], Ruscheweyh derivative operator [7], fractional calculus operator [8], Carlson-Shaffer operator [9], Dziok-Srivatsava operator [10], and also the operator introduced by Abubaker and Darus [11].

**Definition 1**

For 0 ≤ *α* ≤ 1, we let *A* be the subclass of *𝒜*(*n*) consisting of functions of the form (1) that satisfy

(16) |1b(z[𝒟λ,μm(α1,β1)f(z)]′×((1−α)𝒟λ,μm(α1,β1)f(z)+αz[𝒟λ,μm(α1,β1)f(z)]′)−1)|<γ,

where *z* ∈ Δ, *b* ∈ *ℂ*∖{0}, 0 < *γ* ≤ 1, and *𝒟*_*λ*,*μ*_^*m*^(*α*_1_, *β*_1_)*f*(*z*) are as given in (15).

**Definition 2**

For 0 ≤ *α* ≤ 1 we let *B* be the subclass of *𝒜*(*n*), consisting of functions of the form (1) that satisfy

(17) |1b((1−α)𝒟λ,μm(α1,β1)f(z)z  +α[𝒟λ,μm(α1,β1)f(z)]′−1)|<γ,

where *z* ∈ Δ, *b* ∈ *ℂ*∖{0}, 0 < *γ* ≤ 1, and *𝒟*_*λ*,*μ*_^*m*^(*α*_1_, *β*_1_)*f*(*z*) are as given in (15).

By specializing the parameters involved in the above definitions, we could arrive at several known as well as new classes. For example, by taking *λ* = 1, *μ* = 0, *q* = 2, *s* = 1, *α*_1_ = *β*_1_, and *α*_2_ = 1 and the above classes reduced to

(18) A1={f∈𝒜(n) ∣ |1b(z[𝔻mf(z)]′×((1−α)𝔻mf(z)+αz[𝔻mf(z)]′)−1)|<γ},

where *𝔻* ^*m*^ *f*(*z*) denote the Sălăgean derivative of order *m* given by

(19) 𝔻mf(z)=z−∑k=n+1∞kmakzk,B1={f∈𝒜(n) ∣ |1b((1−α)𝔻mf(z)z+α[𝒟mf(z)]′−1)|<γ}.

Similarly, on taking *q* = 2, *s* = 1, *α*_1_ = *η* − 1, (*η* > −1), *α*_2_ = 1, *β*_1_ = 1, one gets

(20) A2={f∈𝒜(n) ∣ |1b(z[Dmf(z)]′            ×((1−α)Dmf(z)              +αz[Dmf(z)]′)−1)|<γ},

where *D* ^*m*^ *f*(*z*) is the operator introduced and studied by Abubaker and Darus [11] given by

(21) Dmf(z)=z−∑k=n+1∞Ckm(η+k−1k−1)zk,B2={f∈𝒜(n) ∣ |1b((1−α)Dmf(z)z+α[Dmf(z)]′−1)|<γ}.

Further, by taking *m* = 0 in the definition of the classes *A* and *B*, we could arrive at *S*_*n*_(*q*, *s*, *α*, *b*, *γ*) and *R*_*n*_(*q*, *s*, *α*, *b*, *γ*) which were introduced and studied by Murugusundaramoorthy et al. [12].

In this paper, we establish the coefficient inequalities for the classes *A* and *B* and several inclusion relationships involving *n*-*δ* neighborhoods of analytic univalent functions with negative and missing coefficients belonging to these classes.

## 2. Coefficient Inequalities

**Theorem 3**

Let the function *f* ∈ *𝒜*(*n*) as given in (1). Then, *f* ∈ *A* if and only if

(22) $$\begin{array}{c} \overset{\infty }{\underset{k=n+1}{\sum }}\left\{\left[1+\alpha \left(k-1\right)\right]\left(\gamma \left|b\right|-1\right)+k\right\}C_{k}^{m}B_{k}a_{k}\leq \gamma \left|b\right|.‍ \end{array}$$

**Proof**

Let the functions of form (1) belong to the class *A*. Then, in view of (15) and (16), we get

(23) $$\begin{array}{c} \left|\frac{\sum_{k=n+1}^{\infty }\left(\left[1+\alpha \left(k-1\right)\right]-k\right)C_{k}^{m}B_{k}a_{k}z^{k}}{z-\sum_{k=n+1}^{\infty }\left[1+\alpha \left(k-1\right)\right]C_{k}^{m}B_{k}a_{k}z^{k}}\right|<\gamma \left|b\right|,\\ \frac{\sum_{k=n+1}^{\infty }\left(\left[1+\alpha \left(k-1\right)\right]-k\right)C_{k}^{m}B_{k}a_{k}r^{k-1}}{1-\sum_{k=n+1}^{\infty }\left[1+\alpha \left(k-1\right)\right]C_{k}^{m}B_{k}a_{k}r^{k-1}}<\gamma \left|b\right|. \end{array}$$

Letting *r* → 1^−^ through real values, we get the required assertion (22). Conversely, suppose *f*(*z*) satisfies (22), then, in view of (16), consider

(24) |z[𝒟λ,μm(α1,β1)f(z)]′−(1−α)𝒟λ,μm(α1,β1)f(z)−αz[𝒟λ,μm(α1,β1)f(z)]′|  −γ|b||(1−α)𝒟λ,μm(α1,β1)f(z)  +αz[𝒟λ,μm(α1,β1)f(z)]′| =|∑k=n+1∞([1+α(k−1)]−k)CkmBkakzk|  −γ|b||z−∑k=n+1∞[1+α(k−1)]CkmBkakzk| <∑k=n+1∞{([1+α(k−1)](γ|b|−1)+k)}      ×CkmBkak−γ|b| ≤0.

Hence, the result follows.

Similarly, we prove the following.

**Theorem 4**

Let the function *f* ∈ *𝒜*(*n*) be as defined in (1). Then, *f* ∈ *B* if and only if

(25) $$\begin{array}{c} \overset{\infty }{\underset{k=n+1}{\sum }}\left[1+\alpha \left(k-1\right)\right]C_{k}^{m}B_{k}a_{k}\leq \gamma \left|b\right|. \end{array}$$

**Corollary 5**

Let the function *f* ∈ *𝒜*(*n*) as given in (1). Then, *f*(*z*) ∈ *A*_1_ if and only if

(26) $$\begin{array}{c} \overset{\infty }{\underset{k=n+1}{\sum }}\left\{\left[1+\alpha \left(k-1\right)\right]\left(\gamma \left|b\right|-1\right)+k\right\}k^{m}a_{k}\leq \gamma \left|b\right|. \end{array}$$

**Corollary 6**

Let the function *f* ∈ *𝒜*(*n*) be as defined in (1). Then, *f* ∈ *B*_1_ if and only if

(27) $$\begin{array}{c} \overset{\infty }{\underset{k=n+1}{\sum }}\left[1+\alpha \left(k-1\right)\right]k^{m}a_{k}\leq \gamma \left|b\right|. \end{array}$$

**Corollary 7**

Let the function *f* ∈ *𝒜*(*n*) as given in (1). Then, *f* ∈ *A*_2_ if and only if

(28) ∑k=n+1∞{[1+α(k−1)](γ|b|−1)+k}Ckm×(η+k−1k−1)ak≤γ|b|.

**Corollary 8**

Let the function *f*(*z*) ∈ *𝒜*(*n*) be as defined in (1). Then, *f* ∈ *B*_2_ if and only if

(29) $$\begin{array}{c} \overset{\infty }{\underset{k=n+1}{\sum }}\left[1+\alpha \left(k-1\right)\right]C_{k}^{m}\left(\begin{array}{c} \eta +k-1\\ k-1 \end{array}\right)a_{k}\leq \gamma \left|b\right|. \end{array}$$

## 3. Inclusion Relationships

**Theorem 9**

If

(30) $$\begin{array}{c} \delta =\frac{\gamma \left|b\right|\left(1+n\right)}{\left[\left(1+n\alpha \right)\left(\gamma \left|b\right|-1\right)+n+1\right]B_{n+1}C_{n+1}^{m}}\;\;\left(\gamma \left|b\right|>1\right), \end{array}$$

then *A* ⊂ *N*_*n*,*δ*_(*e*).

**Proof**

Let *f* ∈ *A*. Then, in view of (22), we have

(31) Cn+1mBn+1[(1+nα)(γ|b|−1)n+1]∑k=n+1∞ak  ≤γ|b|,

(32) $$\begin{array}{c} \overset{\infty }{\underset{k=n+1}{\sum }}a_{k}\leq \frac{\gamma \left|b\right|}{C_{n+1}^{m}B_{n+1}\left[\left(1+n\alpha \right)\left(\gamma \left|b\right|-1\right)n+1\right]}. \end{array}$$

Consider

(33) Cn+1mBn+1∑k=n+1∞kak ≤γ|b|+(1+nα)(1−γ|b|)Bn+1Cn+1m∑k=n+1∞ak ≤γ|b|+(1+nα)(1−γ|b|)γ|b|(1+nα)(γ|b|−1)+n+1 ≤γ|b|(n+1)(1+nα)(γ|b|−1)+n+1.

Hence,

(34) ∑k=n+1∞kak≤γ|b|(n+1)[(1+nα)(γ|b|−1)+n+1]Bn+1Cn+1m=δ.

Hence, the result follows.

In similar manner, we establish the following result.

**Theorem 10**

If

(35) $$\begin{array}{c} \delta =\frac{\gamma \left|b\right|\left(1+n\right)}{\left(1+n\alpha \right)B_{n+1}C_{n+1}^{m}}, \end{array}$$

then *B* ⊂ *N*_*n*,*δ*_(*e*).

**Corollary 11**

If

(36) $$\begin{array}{c} \delta =\frac{\gamma \left|b\right|\left(1+n\right)}{\left[\left(1+n\alpha \right)\left(\gamma \left|b\right|-1\right)+n+1\right]{\left(n+1\right)}^{m}}, \end{array}$$

then *A*_1_ ⊂ *N*_*n*,*δ*_(*e*).

**Corollary 12**

If

(37) $$\begin{array}{c} \delta =\frac{\gamma \left|b\right|\left(1+n\right)}{\left(1+n\alpha \right){\left(n+1\right)}^{m}}, \end{array}$$

then *B*_1_ ⊂ *N*_*n*,*δ*_(*e*).

**Corollary 13**

If

(38) $$\begin{array}{c} \delta =\frac{\gamma \left|b\right|\left(1+n\right)}{\left[\left(1+n\alpha \right)\left(\gamma \left|b\right|-1\right)+n+1\right]C_{n+1}^{m}\left(\begin{array}{c} \eta +n\\ n \end{array}\right)}, \end{array}$$

then *A*_2_ ⊂ *N*_*n*,*δ*_(*e*).

**Corollary 14**

If

(39) $$\begin{array}{c} \delta =\frac{\gamma \left|b\right|\left(1+n\right)}{\left(1+n\alpha \right)C_{n+1}^{m}\left(\begin{array}{c} \eta +n\\ n \end{array}\right)}, \end{array}$$

then *B*_2_ ⊂ *N*_*n*,*δ*_(*e*).

## 4. Neighborhoods for *A* ^*σ*^ and *B* ^*σ*^

In this section, we determine the neighborhood properties of *A* ^*σ*^ and *B* ^*σ*^. Here, the classes *A* ^*σ*^ consist of functions *f* ∈ *𝒜*(*n*) for which there exists a function *g*(*z*) ∈ *A* such that

(40) $$\begin{array}{c} \left|\frac{f\left(z\right)}{g\left(z\right)}-1\right|<1-\sigma \;\left(z\in \Delta ,0\leq \sigma <1\right). \end{array}$$

In the same way, we define *B* ^*σ*^, consisting of functions *f*(*z*) ∈ *𝒜*(*n*) for which there exists another function *g*(*z*) ∈ *B* such that

(41) $$\begin{array}{c} \left|\frac{f\left(z\right)}{g\left(z\right)}-1\right|<1-\sigma \;\left(z\in \Delta ,0\leq \sigma <1\right). \end{array}$$

**Theorem 15**

If *g* ∈ *A* and

(42) σ=1−δn+1×{[(1+nα)(γ|b|−1)+n+1]×Bn+1Cn+1m×([(1+nα)(γ|b|−1)+n+1]×Bn+1Cn+1m−γ|b|)−1}(γ|b|>1),

then *N*_*n*,*δ*_(*g*) ⊂ *A* ^*σ*^.

**Proof**

Suppose *f* ∈ *N*_*n*,*δ*_(*g*), then

(43) $$\begin{array}{c} \overset{\infty }{\underset{k=n+1}{\sum }}k\left|a_{k}-b_{k}\right|\leq \delta ,\\ \overset{\infty }{\underset{k=n+1}{\sum }}\left|a_{k}-b_{k}\right|\leq \frac{\delta }{n+1}. \end{array}$$

Since *g* ∈ *A*, we have

(44) $$\begin{array}{c} \overset{\infty }{\underset{k=n+1}{\sum }}b_{k}\leq \frac{b\left|k\right|}{\left[\left(1+n\alpha \right)\left(\gamma \left|b\right|-1\right)+n+1\right]B_{n+1}C_{n+1}^{m}}. \end{array}$$

Consider

(45) |f(z)g(z)−1|<∑k=n+1∞|ak−bk|1−∑k=n+1∞bk≤δn+1{[(1+nα)(γ|b|−1)+n+1]Bn+1Cn+1m[(1+nα)(γ|b|−1)+n+1]Bn+1Cn+1m−γ|b|}=1−σ.

Therefore, *f* ∈ *A* ^*σ*^ for *σ* given by (42).

**Theorem 16**

If *g* ∈ *B* and

(46) $$\begin{array}{c} \sigma =1-\frac{\delta }{n+1}\left\{\frac{\left(1+n\alpha \right)B_{n+1}C_{n+1}^{m}}{\left(1+n\alpha \right)B_{n+1}C_{n+1}^{m}-\gamma \left|b\right|}\right\}, \end{array}$$

then *N*_*n*,*δ*_(*g*) ⊂ *B* ^*σ*^.

**Corollary 17**

If *g* ∈ *A*_1_ and

(47) σ=1−δn+1{[(1+nα)(γ|b|−1)+n+1](n+1)m×([(1+nα)(γ|b|−1)+n+1]×(n+1)m−γ|b|)−1}(γ|b|>1),

then *N*_*n*,*δ*_(*g*) ⊂ *A*_1_^*σ*^.

**Corollary 18**

If *g* ∈ *B*_1_ and

(48) $$\begin{array}{c} \sigma =1-\frac{\delta }{n+1}\left\{\frac{\left(1+n\alpha \right){\left(n+1\right)}^{m}}{\left(1+n\alpha \right){\left(n+1\right)}^{m}-\gamma \left|b\right|}\right\}, \end{array}$$

then *N*_*n*,*δ*_(*g*) ⊂ *B*_1_^*σ*^.

**Corollary 19**

If *g* ∈ *A*_2_ and

(49) σ=1−δn+1{[(1+nα)(γ|b|−1)+n+1](η+nn)Cn+1m×([(1+nα)(γ|b|−1)+n+1]×(η+nn)Cn+1m−γ|b|)−1}(γ|b|>1),

then *N*_*n*,*δ*_(*g*) ⊂ *A*_2_^*σ*^.

**Corollary 20**

If *g* ∈ *B*_2_ and

(50) $$\begin{array}{c} \sigma =1-\frac{\delta }{n+1}\left\{\frac{\left(1+n\alpha \right)\left(\begin{array}{c} \eta +n\\ n \end{array}\right)C_{n+1}^{m}}{\left(1+n\alpha \right)\left(\begin{array}{c} \eta +n\\ n \end{array}\right)C_{n+1}^{m}-\gamma \left|b\right|}\right\}, \end{array}$$

then *N*_*n*,*δ*_(*g*) ⊂ *B*_2_^*σ*^.
